# Peribiliary Glands as the Cellular Origin of Biliary Tract Cancer

**DOI:** 10.3390/ijms19061745

**Published:** 2018-06-12

**Authors:** Hayato Nakagawa, Yuki Hayata, Tomoharu Yamada, Satoshi Kawamura, Nobumi Suzuki, Kazuhiko Koike

**Affiliations:** Department of Gastroenterology, The University of Tokyo, 7-3-1 Hongo, Bunkyo-ku, Tokyo 113-8655, Japan; hayatayk728@gmail.com (Y.H.); tyism123@gmail.com (T.Y.); shisato13@gmail.com (S.K.); nobu3szk@gmail.com (N.S.); kkoike-tky@umin.ac.jp (K.K.)

**Keywords:** peribiliary gland, cholangiocarcinoma, interleukin (IL)-33, intraductal papillary neoplasm of biliary duct, mouse model, stem cell

## Abstract

The identification of the cellular origin of cancer is important for our understanding of the mechanisms regulating carcinogenesis, thus the cellular origin of cholangiocarcinoma (CCA) is a current topic of interest. Although CCA has been considered to originate from biliary epithelial cells, recent studies have suggested that multiple cell types can develop into CCA. With regard to the hilar and extrahepatic bile ducts, peribiliary glands (PBGs), a potential stem cell niche of biliary epithelial cells, have attracted attention as the cellular origin of biliary tract cancer. Recent histopathological and experimental studies have suggested that some kinds of inflammation-induced CCA and intraductal papillary neoplasms of the bile duct are more likely to originate from PBGs. During inflammation-mediated cholangiocarcinogenesis, the biliary epithelial injury-induced regenerative response by PBGs is considered a key process. Thus, in this review, we discuss recent advances in our understanding of cholangiocarcinogenesis from the viewpoint of inflammation and the cellular origin of CCA, especially focusing on PBGs.

## 1. Introduction

Cholangiocarcinoma (CCA) is a highly malignant tumor exhibiting feature of biliary epithelial differentiation. CCA is classified, according to its anatomical location, as intrahepatic CCA (iCCA), perihilar CCA (pCCA), or distal CCA (dCCA). pCCA and dCCA are distinguished by the insertion site of the cystic duct, and iCCA is defined as CCA arising from the second-order bile duct in the liver [1,2]. Further classification of CCA is based on tumor morphology. The iCCAs are classified into mass-forming, periductal-infiltrating, and intraductal growth, and pCCAs/dCCAs are classified into flat- and nodular-infiltrating, and papillary types [3]. Although surgical resection is the sole potentially curative treatment, regardless of anatomical location and morphological subtype, only 20–30% of CCA patients are amenable to surgical resection because CCA is usually diagnosed at an advanced stage. In addition, the efficacies of other treatment options, which include radiation and chemotherapy, are limited, and there is presently no approved molecular-targeted therapy for CCA [4,5]. Therefore, understanding the molecular mechanisms and the unique pathogenic biology of CCA is an imperative issue.

The identification of the cellular origin of cancer is important for our understanding of the mechanisms regulating cancer. Cancer originates from a normal cell that accumulates genetic mutations and undergoes transformation [6]. Many studies using mouse models have identified tissue-resident, long-lived stem cells in various organs and suggested that these cells are the main cellular origin of cancer [7]. A well-characterized example of this concept is intestinal carcinogenesis originating from the intestinal crypt stem cells [8]. However, the location of tissue-resident adult stem cells has not been definitively determined in the biliary tract. Notably, although biliary tract cancer has been considered to originate from biliary epithelial cells (BECs), recent mouse genetic lineage-tracing experiments have suggested that multiple cell types, including mature hepatocytes, can give rise to iCCA [9,10]. Regarding pCCA/dCCA, anatomical and immunohistochemical analyses have revealed that peribiliary glands (PBGs), clusters of epithelial cells residing in the submucosal compartment of extrahepatic and large intrahepatic bile ducts (EHBDs and IHBDs, respectively), are potential stem/progenitor cell niches of BECs; thus, PBGs have attracted attention as a potential origin of pCCA/dCCA [11].

Although a mouse disease model is a powerful tool to investigate the cellular origin of cancer, there has not been a good mouse model for extrahepatic CCA. Recently, we established a new mouse model of extrahepatic CCA whose malignant progression depends on biliary epithelial injury and inflammation. This mouse model has suggested that PBGs are the cellular origin of extrahepatic CCA [12]. Thus, in this review, we first provide a brief overview of CCA, and then discuss recent advances in the understanding of cholangiocarcinogenesis from the viewpoint of the inflammation and cellular origin of CCA, especially focusing on PBGs.

## 2. Chronic Inflammation Is Key in the Epidemiology and Risk Factors of CCA

The global incidence of CCA varies widely and is highest in Southeast Asia, China, and the Republic of Korea, which is approximately 10-fold higher than that of the United States or Europe [2,13]. Such a wide geographical variation in the incidence of CCA is mostly attributed to the prevalence of risk factors, including liver fluke, hepatitis viral infection, primary sclerosing cholangitis (PSC), hepatolithiasis, and congenital abnormalities of the pancreatic and biliary ducts [1]. Infection with liver flukes, *Opisthorchis viverrini* and *Clonorchis sinesis*, is highly prevalent in East Asia, and a meta-analysis revealed its strong association with CCA (odds ratio (OR), 4.8; 95% confidence interval (CI), 2.8–8.4) [14]. Chronic infection with hepatitis B virus (HBV) or hepatitis C virus, which is also common in Asian countries, increases the risk of iCCA (OR, 2.6, 95% CI, 1.5–4.6; OR, 1.8, 95% CI, 1.4–2.4, respectively). PSC is a well-established risk factor, especially in Western countries, with a lifetime risk of CCA estimated as 7–14% [15,16,17,18]. Importantly, most of these conditions create chronic inflammation in the biliary tree and liver, indicating that chronic inflammation plays a key role in cholangiocarcinogenesis.

Although Virchow first suggested the relationship between inflammation and cancer in the 19th century, the molecular mechanisms have been gradually identified during the last 10–20 years [19]. The mechanism of hepatocellular carcinoma (HCC), the other type of liver cancer, is one of the most extensively investigated inflammation-based carcinogenic processes, because more than 90% of HCCs develop in the context of chronic liver damage and inflammation [20]. Chronic liver damage induces a persistent cycle of necroinflammation and hepatocyte regeneration, resulting in genetic mutations in hepatocytes and expansion of initiated cells, eventually leading to HCC. During these processes, several signaling pathways, such as the nuclear factor-κB (NF-κB) and STAT3 pathways, and inflammatory cytokines, such as tumor necrosis factor (TNF)-α and interleukin (IL)-6, play important roles in cellular autonomous and non-autonomous behaviors [21,22,23]. Furthermore, recent studies regarding gastrointestinal tract cancer have reported that chronic epithelial injury accompanied by inflammation induces expansion of stem cell niches, leading to genetic and epigenetic alterations in stem/progenitor cells, eventually resulting in invasive cancer [7]. These findings suggested that chronic epithelial injury and inflammation might accelerate tissue-resident stem/progenitor cell-derived carcinogenesis. Although similar theories have been proposed for inflammation-related cholangiocarcinogenesis, the exact mechanisms and cellular origins in cholangiocarcinogenesis have remained unclear. However, several recent studies have shed some light on this intriguing and unsolved problem, as discussed in later sections.

## 3. Mutation Spectrum of CCA

Before discussing the mechanisms and cellular origins of CCA, recent advances in CCA genomics should be mentioned. Next-generation sequencing technology has enabled comprehensive mutational and transcriptome profiling of CCA and five identified core driver mutations (*TP53*, *KRAS*, *SMAD4*, *ARID1A*, and *BAP1*) and five molecular modules (kinase-RAS, TGFβ-SWI/SNF-MYC, TP53, RB-cell cycle, and epigenetic regulators) [24,25]. Epigenetic regulators include *IDH1/2*, *BAP1*, methylcytosine dioxygenase family members (*TET1*, *TET2*, and *TET3*), and histone demethylases and methylases (*MLL2* and *MLL3*). In addition, some molecular alterations characteristics of CCA have been identified, such as fusion transcripts of *FGFR2* and the protein kinase A (PKA) pathway (*PRKACA* or *PRKACB*) [24,26]. However, the frequency of each genetic alteration is not high and even mutated *TP53*, the most commonly mutated gene, is present only in approximately 35% of CCA patients, indicating that CCA is a heterogeneous cancer.

*IDH1/2* and *BAP1* are frequently mutated in liver fluke-related CCA, whereas *TP53* mutations show a reciprocal pattern [27]. Furthermore, the frequencies of *KRAS* and *IDH1/2* mutations are significantly low in hepatitis virus-related iCCA when compared with hepatitis virus-unrelated iCCA [28,29]. In contrast, *TERT* promoter hotspot mutations are strongly associated with hepatitis virus-related iCCA [29]. These findings indicated that underlying etiologies influence the mutational profile of CCA. In addition, anatomical location is also an important determinant for genetic alterations in CCA. *FGFR2* fusions and *IDH1/2* mutations occur exclusively in iCCA, and *BAP1* is frequently mutated in iCCA when compared with extrahepatic CCA and gallbladder cancer. However, fusion of *PRKACA* or *PRKACB* and mutations of *ELF3* predominantly occur in extrahepatic CCA [24]. These findings suggest that the carcinogenesis process may differ according to the anatomical subtypes and underlying etiologies of CCA. Although the same chemotherapeutic strategy is presently used for CCA, irrespective of these factors, more personalized approaches should be explored. For example, clinical trials of kinase inhibitors targeting *FGFR2* are ongoing. The response percentage of a recent phase 2 clinical trial was 18.8% for *FGFR2* fusion-positive CCA; however, acquired resistant mutations to FGFR inhibition were identified [30].

## 4. Cellular Origins of iCAA

The biliary tree is grossly divided into IHBDs and EHBDs, and IHBDs are subdivided into large and small IHBDs [31]. Area ducts, segmental ducts, and right/left hepatic ducts are classified as large IHBDs, while interlobular bile ducts and septal ducts are classified as small IHBDs. The canals of Hering connect the proximal end of small IHBDs to bile canaliculi that are formed by the apical membrane of adjacent hepatocytes. These classifications are important because BECs of EHBDs and large IHBDs differ functionally and morphologically from those of small IHBDs. In addition, in the embryonic stage, EHBDs develop from hepatic endodermal cells in the caudal region of embryonic hepatic diverticula, while IHBDs originate from periportal hepatoblasts, forming ductal plates that originate from the cranial part of hepatic diverticula [32,33,34]. Therefore, small IHBDs, large IHBDs, and EHBDs may exhibit distinct properties and different carcinogenetic processes. Furthermore, based on the anatomical location and developmental processes of the liver, adult liver stem/progenitor cells have been suggested to reside in the canals of Hering [35], so these cells may be a potential origin of liver cancer, including iCCA.

Genetic lineage-tracing studies in mice facilitating the conditional expression of a reporter gene in a targeted cellular population have led to a number of significant advances in our understanding of tissue homeostasis and the cellular origins of cancer [36]. Sekiya and Suzuki investigated the cellular origin of iCCA using a mouse model of thioacetamide (TAA)-induced iCCA in combination with genetic lineage-tracing methods [9]. *Rosa26-Lox-Stop-Lox(LSL)-LacZ* reporter mice were crossed with *Albumin^CreERT^* mice or *K19^CreERT^* mice, in which hepatocytes and BECs (including cells in the canals of Hering) were genetically labeled in a cell type specific manner by tamoxifen (TAM) administration. Surprisingly, iCCAs were derived from hepatocytes rather than from BECs in this model. Chronic liver injury caused by continuous administration of TAA induced conversion of hepatocytes into ductal cells through Notch activation, and eventually led to iCCAs. Another study also suggested the possibility of mature hepatocytes as the cellular origin of iCCA, by introducing hepatocyte specific activation of Akt and Notch pathways by hydrodynamic tail vein injection in combination with genetic lineage-tracing methods [10]. Importantly, because liver injury has been known to induce ductular metaplasia of hepatocytes [37,38,39], chronic liver injury-related iCCAs may be more prone to originate from hepatocytes through ductular metaplasia. Whether iCCAs originate from hepatocytes in humans remains unknown, but there have been some suggestive findings. First, HBV genome integration was detected in HBV-related iCCAs [24,29]. Because HBV shows strong tropism for hepatocytes, these iCCAs may be derived from HBV-infected hepatocytes. Second, mutation profiles in hepatitis-positive iCCAs were similar to those of HCCs, but distinct from hepatitis-negative iCCAs [29], suggesting that chronic hepatitis-related iCCAs and HCCs may arise from the same cellular origins, including mature hepatocytes or liver stem/progenitor cells. Third, intracytoplasmic p62-positive hyaline bodies that are specific for damaged hepatocytes are commonly seen in hepatitis-related iCCAs [40]. Thus, chronic liver injury-mediated ductular metaplasia of hepatocytes may also be involved in human iCCAs.

In contrast, some studies reported that iCCAs originated from BECs. Mice with duct cell specific Kras activations and phosphatase and tensin homologue (PTEN) deletions generated by crossing *K19^CreERT^* mice with *LSL-Kras^G12D^* and *Pten^flox/flox^* mice developed iCCAs, while mice with hepatocyte specific Kras activations and PTEN deletions generated using *Albumin^CreERT^* mice developed HCCs [41], suggesting that iCCAs and HCCs are derived from BECs and hepatocytes, respectively. In addition, mice with duct cell specific deletion of p53 generated by crossing *K19^CreERT^* mice with *Tp53^flox/flox^* mice in combination with chronic TAA administration developed iCCAs from BECs [42]. Taken together, both hepatocytes and BECs may be cellular origins of iCCAs in a context-dependent manner (Table 1).

## 5. PBGs Are Possible Stem/Progenitor Niches for BECs

Next, we review the recent advances in the understanding of stem/progenitor cells and carcinogenesis in large IHBDs and EHBDs. Tubuloalveolar glands with mucinous and serous acini, so-called PBGs, are distributed around large IHBDs and EHBDs. PBGs can be further classified into intramural and extramural glands based on the distance from the bile duct lumen. PBGs are predominantly located at branching points of the biliary tree, such as the hilar bile duct, cystic duct, and periamupullary region, and they connect to the bile duct lumen through small canals [11]. Although PBGs were identified by anatomists in the 19th century, their pathophysiological role has not been definitively identified [31].

Recent studies have suggested the presence of mutipotent adult stem/progenitor cells in human PBGs. Although the majority of cells in PBGs are sero-mucinous epithelial cells and are considered to modulate bile composition by secreting serous and mucinous components, some populations were found to express endodermal stem/progenitor cell markers, such as PDX1, Sox9, and Sox17, which are transcription factors functionally involved in the development of the pancreatico-biliary system [43,44,45]. PBGs also express several other stem/progenitor cell markers (Table 2), and importantly, these markers are also known as cancer stem cell markers [46,47]. Isolated cells from PBGs could clonogenically expand in vitro and differentiate into hepatocytes, cholangiocytes, and pancreatic β-cells, and these cells could be transplanted into the liver of immunocompromised mice and function as differentiated hepatocytes and cholangiocytes [48]. Immunohistochemical analyses showed that cells with stem/progenitor properties appeared to be located at the bottom of PBGs near the fibromuscular layer [47]. A recent anatomical study performing three-dimensional reconstruction of mouse EHBDs showed that PBGs elongate to form and elaborate a peribiliary network with the EHBD wall, and could proliferate in response to biliary epithelial injury such as rhesus rotavirus infection and ligation of the common bile duct [49]. In addition, significant proliferation and hyperplasia of PBGs were observed in patients with various hepatobiliary diseases, such as bacterial cholangitis, PSC, liver fluke infection, hepatolithiasis, and cirrhosis [50,51,52,53]. Furthermore, injury of PBGs was strongly associated with the occurrence of biliary structures following liver transplantation [54]. Notably, cells in PBGs responded to diabetes with proliferation and differentiation toward insulin-producing cells in human and rodents [55]. Based on these findings, biliary tree stem/progenitor cells have been considered to reside in PBGs that may participate in the renewal of surface BECs by supplying mature cholangiocytes from deep to the surface side, as is the case in intestinal crypts [11]. Accordingly, PBGs can also be a potential cellular origin of pCCA/dCCA.

## 6. A Mouse Model Suggests PBGs Are the Cellular Origin of CCA

Because the lack of an appropriate mouse model has hampered the investigation of extrahepatic CCA, we tried to establish a new mouse model. We first generated mice with TAM-inducible duct cell specific Kras activation and TGFβR2 deletion by crossing *LSL-Kras^G12D^*, *Tgfbr2^flox/flox^*, and *K19^CreERT^* mice (*KT-K19^CreERT^*) based on the frequent alterations of Ras and TGFβ/SMAD signaling pathways in human CCA. However, *KT-K19^CreERT^* mice only developed mild hyperplasia in the EHBDs. Surprisingly, additional deletion of the adhesion molecule, E-cadherin, by crossing *KT-K19^CreERT^* mice with *Cdh1^flox/flox^* mice (*KTC-K19^CreERT^*), rapidly induced invasive extrahepatic CCA within four weeks of TAM administration [12]. Histologically, moderately to poorly differentiated adenocarcinoma cells expanded along the EHBD wall (so-called periductal infiltration) and extended to the intrahepatic hilar area, including the large IHBDs. In contrast, the peripheral small IHBDs revealed only dysplastic changes, but did not develop peripheral type iCCA, despite a similar gene recombination rate among parts of the biliary tree (approximately 40%). Furthermore, although CCA extended to the cystic duct, the gallbladder was almost intact. Thus, the sensitivity to these mutations differed depending on the location in the biliary tree.

E-cadherin is the core protein connecting the epithelial adherens junctions with neighboring cells, whose loss is associated with poor prognosis in various cancers, including CCA [56,57]. Additionally, there is growing evidence that dysregulation of E-cadherin and its related protein-mediated cell–cell junctions can cause biliary tract diseases in mice. We previously reported that liver specific E-cadherin knockout mice, generated by crossing *Cdh1^flox/flox^* mice with *Albumin-Cre* mice, developed sclerosing cholangitis due to an impaired intrahepatic biliary network, and induced liver tumors with an epithelial–mesenchymal transition (EMT) phenotype when crossed with *LSL-Kras^G12D^* mice [58]. Mice with liver specific deletion of α-catenin, β-catenin, or p120 catenin, which form adherens junction complexes with E-cadherin, have also been shown to develop cholestatic liver diseases [59,60,61]. Importantly, E-cadherin expression in human BECs typically exhibits a clear membranous pattern, whereas BECs in patients with PSC have fragmented and cytoplasmic expression patterns of E-cadherin [62]. A recent study showed that lysyl oxidase-like protein 2 (LOXL2) produced by reactive BECs, portal myofibroblasts, and Kupffer cells repressed E-cadherin expression and disrupted the barrier function of BECs through Snail activation, resulting in aggravation of cholestatic liver injury in mice [63]. An inverse correlation between LOXL2 and E-cadherin expression was confirmed in medium-sized bile ducts from PSC patients. These findings indicated the importance of E-cadherin as a guardian to maintain the homeostasis of biliary systems, which is the reason we deleted E-cadherin in *KT-K19^CreERT^* mice.

**Figure 1:**
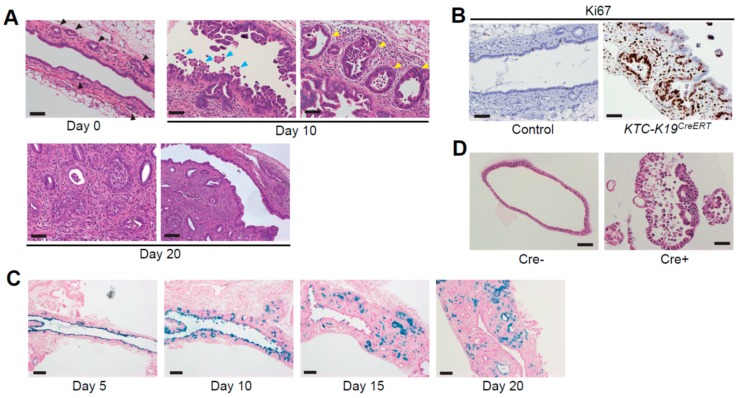
Histological findings of extrahepatic bile ducts (EHBDs) in *KTC-K19^CreERT^* mice. (**A**) Hematoxylin and eosin (H&E)-stained images of EHBDs from *KTC-K19^CreERT^* mice at the indicated time points following tamoxifen (TAM) administration (scale bar: lower right panel, 100 μm; others, 50 μm). *KTC-K19^CreERT^* mice were orally administered 200 mg/kg TAM for three consecutive days and sacrificed at the indicated time points. Black arrowheads, normal peribiliary glands (PBGs); blue arrowheads, biliary epithelial cells (BECs) detaching from the bile duct epithelium; yellow arrowheads, enlarged and dysplastic PBGs; (**B**) Ki67 immunostaining of EHBDs from Cre-negative control and *KTC-K19^CreERT^* mice at 10 days following TAM administration (scale bar, 50 μm); (**C**) LacZ-stained images of EHBDs of *KTC-LacZ-K19^CreERT^* mice at the indicated time points following TAM administration (scale bar, 200 μm). LacZ expression was detected by staining with X-gal, as described previously [12]. LacZ-positive BECs were detached from the bile duct epithelium, and subsequently luminal surface BECs were replaced by LacZ-negative cells. However, LacZ-positive cells remained in the PBGs, and eventually LacZ-positive cancer glands spread through the subepithelial area; (**D**) H&E-stained images of EHBD organoids from Cre-negative *KTC* mice infected with Cre-expressing or control lentivirus (scale bar: 50 μm). To induce recombination, organoids were infected with Cre-expressing or control lentivirus, and transduced cells were selected using puromycin, as described previously [12].

In *KTC-K19^CreERT^* mice, E-cadherin deletion led to loss of cell–cell adhesion of BECs in EHBDs, which resulted in detachment of BECs from the EHBD wall (Figure 1A). The epithelial defect caused by E-cadherin deletion subsequently induced inflammation and a regenerative response by PBGs (Figure 1B). However, because PBGs supplied the niche where cells harboring mutations could survive, the regenerative response to biliary injury resulted in promotion of CCA development from PBGs. Time course analyses of EHBD tissue revealed that PBGs gradually became enlarged and morphologically dysplastic, accompanied by mitotic figures during this process (Figure 1A). Genetic lineage tracing experiments by crossing with *Rosa26-LSL-LacZ* reporter mice (*KTC-LacZ-K19^CreERT^*) also suggested PBGs as the cellular origin of CCA (Figure 1C), and indeed, the distribution of CCA in *KTC-K19^CreERT^* mice largely corresponded with the distribution of PBGs (i.e., EHBDs and perihilar large IHBDs). In humans, proliferation and hyperplasia of PBGs were observed in patients with various types of biliary diseases as mentioned above [50,51,52,53]. An extensive histological analysis of human livers identified early and pre-invasive lesions of CCAs (biliary intraepithelial neoplasms) in PBGs [64]. PBGs of patients with hepatolithiasis frequently harbor Kras mutations [65], and PBGs in PSC patients express the DNA damage marker γH2AX and EMT markers, such as Snail and α-SMA, accompanied by activation of Sonic Hedgehog (Shh) signaling, as is the case in *KTC-K19^CreERT^* mice [51]. Inhibition of Shh signaling was shown to suppress CCA development in experimental models [66]. Thus, *KTC-K19^CreERT^* mice mimic the carcinogenesis process of chronic biliary injury-related CCAs in humans, and biliary injury-related CCAs are more likely to originate from PBGs. In addition, CCA arising from PBGs may tend to spread through the PBG network, which leads to periductal infiltration. Consistent with this possibility, a recent detailed histological study identified the periductal spread of pCCA through the PBG network in humans [67].

## 7. Interleukin (IL)-33 Promotes Proliferation of PBGs and Development of CCAs

Death-driven compensatory proliferation to repair tissue defects promotes inflammation-associated carcinogenesis though production of various tumor-promoting cytokines and chemokines that can stimulate proliferation and survival of premalignant cells [68]. We recently established a method of biliary organoid culture from mouse EHBDs following gene recombination using a lentivirus expressing Cre-recombinase, which can recapitulate loss of E-cadherin-induced shedding of BECs and form subcutaneous tumors in mice (Figure 1D). Using these organoids, we performed transcriptome analyses and identified IL-33 as a key factor linking biliary epithelial injury, regeneration, and cholangiocarcinogenesis [12]. IL-33 is a member of the IL-1 family, originally described as an inducer of type 2 innate immunity in parasitic infections or allergies [69], and it was recently reported to promote BEC proliferation, particularly in EHBDs through activation of type 2 innate lymphoid cells (ILC2s) [70]. Importantly, IL-33 functions as an “alarmin”, released from dying or damaged cells due to infection or tissue injury, and subsequently induces a Th2 immune response to not only eliminate pathogens, but also to repair the injured tissue [69,71]. In *KTC-K19^CreERT^* mice, dying or damaged BECs due to loss of E-cadherin release IL-33 as an alarm signal that, in turn, stimulates regeneration by PBGs through secretion of IL-13 and amphiregulin from ILC2, eventually resulting in promotion of cholangiocarcinogenesis from PBGs. Furthermore, exogenous administration of IL-33 to *KT-K19^CreERT^* mice induced CCA development, suggesting that IL-33 cooperates with the effects of Kras and TGFβR2 mutation in the development of CCA. Of note, patients with liver fluke infection also showed significantly increased biliary epithelial IL-33 expression and serum IL-33 levels [72]. Together with our data, IL-33 may be a link between liver fluke infection and CCA development. Blocking IL-33 significantly suppressed CCA development in *KTC-K19^CreERT^* mice, so IL-33 may be a potential therapeutic target for CCA.

IL-33 may also be implicated in the unique distribution of CCA in *KTC-K19^CreERT^* mice, which develop predominantly in EHBDs and perihilar IHBDs. Exogenous administration of IL-33 induced inflammation and BEC proliferation in EHBDs and perihilar large IHBDs, whereas the peripheral small IHBDs and gallbladder were largely unaffected. IL-33-administered *KT-K19^CreERT^* mice also showed a similar distribution of CCA. Although IL-33 was shown to have a direct proliferative effect on cholangiocytes in vitro, this effect was restricted in cells derived from EHBDs, but not from small IHBDs [70]. In human CCAs, IL-33 is predominantly expressed in large-duct iCCAs and pCCAs when compared with small-duct iCCAs [73]. These findings suggested that IL-33 might be more dominantly involved in cholangiocarcinogenesis of pCCA/dCCA. Of note, although administration of IL-33 induced proliferation of both luminal surface BECs and PBGs, Ki67 staining revealed that IL-33 exerted greater effects on proliferation of PBGs when compared with surface BECs [12], suggesting that IL-33 may accelerate CCA development from PBGs.

**Figure 2:**
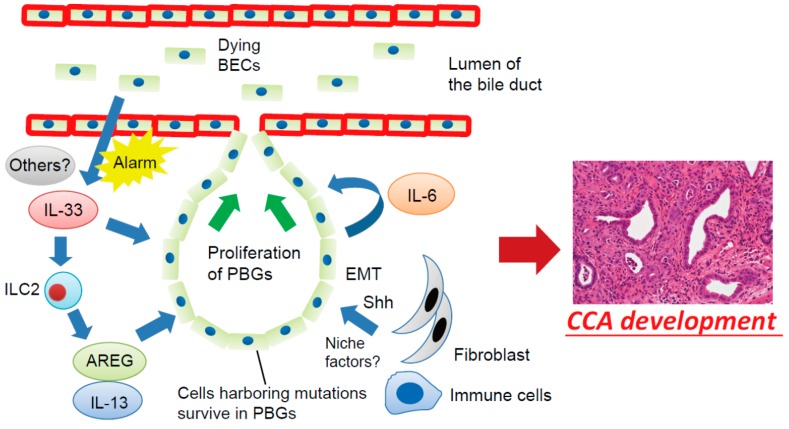
Proposed mechanisms involving the BEC injury-induced regenerative inflammatory response and cholangiocarcinogenesis. Dying or damaged luminal surface BECs release interleukin (IL)-33 and other alarmins to induce regenerative responses by PBGs. IL-33 promotes proliferation of PBGs, directly or indirectly, through activation of autocrine IL-6 signaling or ILC2, which produces effector cytokines and growth factors, such as IL-13 and amphiregulin (AREG). However, cells harboring mutations survived in the PBGs; therefore, the regenerative response to biliary injury resulted in development of cholangiocarcinoma (CCA) from PBGs. During this process, a microenvironment in the PBGs supports cancer-initiating cells in the form of cell–cell contacts and/or secreted factors such as Sonic Hedgehog (Shh) released from surrounding fibroblasts, although the precise mechanisms remain poorly understood. EMT—epithelial–mesenchymal transition.

Yamada et al. showed that transposase-mediated transduction of active Akt and YAP in BECs, coupled with bile duct ligation, followed by IL-33 administration, resulted in CCA in mice [74]. In this study, IL-33 enhanced IL-6 expression in cholangiocytes, which in turn activated STAT3 signaling and facilitated the development of CCA. IL-6/STAT3 signaling is an important pathway in inflammation-associated cancers, such as HCC and colorectal cancer [75,76,77]. A gene expression profiling study of human iCCA identified two main subclasses, the inflammation class and the proliferation class, and activation of the IL-6/STAT3 signaling pathway was the main component of the inflammation class [78]. Mouse CCA induced by activation of Akt and YAP in combination with IL-33 exhibited a similar expression pattern of human CCA [74]. Serum IL-6 levels have been reported as potential biomarkers of CCA [79,80], which further supports the significance of this pathway. Therefore, IL-33-mediated IL-6/STAT3 activation may play a key role in inflammation-associated cholangiocarcinogenesis. Proposed mechanisms involving the BEC injury-induced regenerative inflammatory response and cholangiocarcinogenesis from PBGs are shown in Figure 2.

Very recently, the other alarmin high-mobility group B1 (HMGB1) has been reported to play a key role in chronic liver injury-induced ductular/progenitor cell expansion in the liver (so-called ductular reaction) [81,82]. Liver specific knockout of HMGB1 in mice reduced not only the ductular reaction, but also chronic liver injury or autophagy deficiency-associated HCC development. Therefore, alarmins other than IL-33 may also be implicated in BEC injury-induced regenerative inflammatory responses, and cholangiocarcinogenesis from PBGs.

## 8. PBGs Are the Potential Cellular Origin of Intraductal Papillary Neoplasms of the Biliary Duct

Another type of biliary tumor is biliary cystic neoplasm, which is classified as mucinous cystic neoplasm (MCN) and intraductal papillary neoplasm of the biliary duct (IPNB), according to the 2010 World Health Organization classification [3]. MCN is characterized by mucin-positive lining epithelia associated with ovarian-like stroma in the cyst wall, and occurs almost exclusively in females. MCN usually lacks luminal communication with the bile duct lumen. On the other hand, IPNB shows papillary or villous growth of differentiated neoplastic epithelia and exhibits luminal communication with the bile duct lumen. IPNB is considered to arise from luminal BECs, and affected ducts show cystic dilatation due to mucin-hypersecretion. Based on these findings, IPNB is regarded as a biliary counterpart of intraductal papillary mucinous neoplasms (IPMNs) of the pancreas [83,84]. Although the prognosis of IPNB is better compared with classical CCA, IPNB is also recognized as a precursor of invasive carcinoma.

Recently, several histological studies have suggested not only luminal BECs, but also PBGs as the potential cellular origin of IPNB. Nakanishi et al. first reported a case of surgically resected IPNB potentially originating from PBGs, according to histological findings. This lesion contained an in situ carcinoma at the bottom of the PBGs [85]. They also reported another case of IPNB potentially originating from PBGs and found that the neoplastic epithelia of the tumor expressed MUC6 gastric mucin, which was strongly expressed in PBGs, but not in the epithelia lining bile duct [86], further supporting the possibility of PBGs as the cellular origin of IPNB. Strong expression of MUC6 in IPNB was confirmed by other studies that reported cases of possible PBG-derived IPNBs [87,88]. In the case of IPNB reported by Miyata et al. the cystic tumor did not communicate with the bile duct lumen but lacked features of MCNs, suggesting that communication with the bile duct lumen might be lost during development and progression of IPNB from PBGs [87]. Thus, IPNBs may arise, at least in part, from PBGs. According to the classification of IPMNs, which are classified into the main duct type and branch type, IPNBs arising from PBGs may correspond to the branch type IPMN [85]. Furthermore, IPNBs originating from PBGs have been suggested as preneoplastic lesions of mucin-producing CCAs, which morphologically resemble PBGs [89].

## 9. Future Perspectives

As discussed in this review, increasing focus is being placed on PBGs. Because biliary atresia is a leading cause of pediatric liver transplantation [90], and ischemic stricture of the bile duct is one of the most common complications following liver transplantation [91], understanding the mechanisms responsible for bile duct regeneration will be important for the identification of new therapeutic strategies for these conditions. We recently established an organoid culture system from mouse EHBD, as mentioned above [12], and more recently, bioengineered bile duct-like tubes generated using human EHBD-derived organoids were transplanted into the mouse injured bile duct to repair it [92]. This study is promising in terms of regenerative medicine for biliary diseases and has potential clinical applications. However, cellular and molecular understanding of the endogenenous regenerative mechanism of the bile duct has the same importance as generating a more effective problem solving strategy. In this sense, clarifying whether and how PBGs are implicated in homeostasis and regeneration of the bile duct is mandatory. In addition, recent histopathological and experimental studies have suggested that some chronic inflammation-driven CCAs and IPNBs are likely to originate from PBGs. CCA is one of the most challenging malignancies with poor overall survival; thus, identification of the cellular origin is crucial in enhancing our understanding of the mechanisms regulating the process of cholangiocarcinogenesis. How could the knowledge and insights of PBGs as the cellular origin of biliary tract cancer be transferred for the clinic-pathological evaluation of CCA? For example, to diagnose a malignant biliary structure due to CCA, brushing cytology and intraductal biopsies are usually performed during endoscopic retrograde cholangiography, but their sensitivities are relatively low (approximately 50%) [93]. Such low sensitivities may be attributable to the fact that CCAs originating from PBGs do not appear at the luminal surface, but spread subepithelially as seen in *KTC-K19^CreERT^* mice. In addition, CCAs with periductal infiltration are known to have a poor prognosis with higher rates of recurrence following resection [94]. Therefore, CCAs originating from PBGs may be associated with a worse clinical outcome due to delayed diagnosis and periductal progression. However, although we introduced recent evidence suggesting the possibility of PBGs as a stem/progenitor cell niche of BECs, and suggested the cellular origins of CCA, there is no conclusive evidence at present. To definitively determine these origins, experiments of cell fate mapping for PBGs using PBG specific gene recombination are needed. Because a specific marker of PBGs has not been identified, this remains a subject of future investigation. Furthermore, niches for stem/progenitor cells and tumor-originating cells are generally supported by microenviroments that regulate their stemness by providing cues in the form of cell–cell contacts and/or secreted factors [95]. However, it remains almost completely unknown how such a microenvironment is maintained in PBGs. Therefore, research of PBGs has only begun, and further studies are needed.

## Figures and Tables

**Table 1:** Mouse models for tracing the cellular origin of cholangiocarcinoma (CCA).

Types of CCA	Methods for CCA Induction	Methods for Lineage Tracing	Cellular Origin
Intrahepatic CCA	Chronic thioacetamide (TAA) administration	*Rosa26-LSL-LacZ* mice crossed with *AlbuminCre^ERT^* mice or *K19Cre^ERT^* mice	Hepatocytes
	Hepatocyte-specific activation of Akt and Notch pathways by hydrodynamic tail vein injection	Injection of adenoassociated virus serotype 8 vector expressing Cre from transthyretin promoter into *Rosa26-LSL-EYFP* mice	Hepatocytes
	Duct cell-specific Kras activation and PTEN deletion	*K19Cre^ERT^* mice crossed with *LSL-Kras^G12D^* mice and *Pten^flox/flox^* mice	Cholangiocytes
	Duct cell-specific p53 deletion in combination with chronic TAA administration	*K19Cre^ERT^* mice crossed with *p53^flox/flox^* mice and *Rosa26-LSL-EYFP* mice	Cholangiocytes
Extrahepatic CCA	Duct cell-specific activation of Kras and deletion of TGFβR2 and E-cadherin	*K19Cre^ERT^* mice crossed with *LSL-Kras^G12D^*, *Tgfbr2^flox/flox^*, *CDH1^flox/flox^*, and *Rosa26-LSL-LacZ* mice	Peribiliary glands (PBGs)

**Table 2:** Stem/progenitor cell markers expressed in PBGs.

Types of Stem/Progenitor Cell Markers	Stem/Progenitor Cell Markers Expressed in PBGs
Pluripotency genes	Oct4, Nanog
Stem cell surface markers	CD133, CXCR4, CD44
Markers of endodermal stem cells	Pdx1, Sox9, Sox17, Foxa2
Markers of hepatic stem cells	EpCAM, NCAM
Markers of intestinal stem cells	Lgr5
